# „Frailty“ bei nephrologischen Erkrankungen

**DOI:** 10.1007/s00391-021-01953-0

**Published:** 2021-08-05

**Authors:** Lena Schulte-Kemna, Miriam Künzig, Dhayana Dallmeier, Michael Denkinger, René van Erp, Martin Kächele, Jochen Klaus, Bernd Schröppel

**Affiliations:** 1 Klinik für Innere Medizin I, Sektion Nephrologie, Universitätsklinikum Ulm, Albert-Einstein-Allee 23, 89081 Ulm, Deutschland; 2 AGAPLESION Bethesda, Institut für Geriatrische Forschung der Universität Ulm und Geriatrisches Zentrum Ulm/Alb-Donau, Ulm, Deutschland; 3 Dept. of Epidemiology, Boston University School of Public Health, Boston, USA; 4 Klinik für Innere Medizin I, Universitätsklinikum Ulm, Ulm, Deutschland

**Keywords:** Frailty, Gebrechlichkeit, Dialyse, Niere, Geriatrie, Frailty, Infirmity, Dialysis, Kidney, Geriatric medicine

## Abstract

Frailty beschreibt einen Zustand reduzierter Belastbarkeit gegenüber Stressfaktoren, welcher u. a. Folge altersbedingter Abbauprozesse verschiedener Organsysteme ist und mit einem erhöhten Risiko für Stürze, Hospitalisationen, funktionelle Einschränkung und Mortalität assoziiert ist. Frailty tritt bei Patienten mit CKD früher und häufiger auf als in der Allgemeinbevölkerung und ist ein wichtiger und potenziell modifizierbarer Risikofaktor für eine erhöhte Sterblichkeit und Hospitalisationsrate sowie eine reduzierte Lebensqualität. Die Erkennung von Frailty bei Patienten mit CKD ermöglicht eine Identifikation von wichtigen und potenziell modifizierbaren Risikofaktoren.

Eine frühzeitige nephrologische Beurteilung sowie eine interdisziplinäre Zusammenarbeit mit Geriatern, Allgemeinmedizinern, Physiotherapeuten, Ergotherapeuten und Ernährungsberatung sind essenzielle Bausteine in der Prävention und Therapie von Frailty.

Zur Messung von Frailty existieren verschiedene Instrumente, wobei keines in der Erkennung von Frailty bei CKD-Patienten als überlegen identifiziert wurde. Die Auswahl des Screeninginstruments sollte daher unter Berücksichtigung der klinischen Rahmenbedingungen und der verfügbaren Ressourcen erfolgen. In diesem Artikel wird eine Strategie zur frühen Identifikation von gebrechlichen Patienten mit CKD vorgeschlagen, welche auf einer multidiemensionalen und interdisziplinären Einschätzung beruht. Wichtiger als die Methode ist jedoch die Notwendigkeit, Patienten mit Frailty zu identifizieren.

## Abstract (en)

The term frailty describes a complex syndrome of reduced resistance to stress factors as a consequence of age-related degeneration in various organ systems.

In the general population frailty is associated with poor clinical outcomes, including an increased risk of falls, hospitalization, functional impairment and mortality. Frailty occurs earlier and its prevalence is higher in patients with chronic kidney disease (CKD) compared to the general population. Frail patients with CKD, on dialysis or not, have reduced quality of life and increased hospitalization and mortality rates, regardless of age, sex or comorbidities.

The identification of frailty in patients with CKD can lead to the detection of important and potentially modifiable risk factors. Early nephrological evaluation coupled with an interdisciplinary approach including primary care physicians, geriatricians, physiotherapists, occupational therapists and nutritionists, is fundamental in the prevention of frailty as well as in the management of frail patients with CKD.

Several instruments have been developed to screen for and assess the degree of frailty; however, there is currently no recommendation as to which should be used in nephrology and how to manage frail patients with CKD. In this article we suggest an approach based on a multidimensional, interdisciplinary evaluation aimed at the early identification and management of frail CKD patients independent of the clinical setting of admission; however, more important than the method used is the need to identify and follow-up on frail CKD patients.

## Einführung

Die Prävalenz der chronischen Nierenerkrankungen (CKD) steigt mit zunehmendem Alter. Das Projekt „Global Burden of Disease“ schätzt eine weltweite Zunahme der CKD um 19,6 % zwischen 2005 und 2015, wobei dies im Wesentlichen auf die Alterung der Bevölkerung zurückgeführt wird [1]. Neben einem erhöhten Risiko für Mortalität, kardiovaskuläre und metabolische Komplikationen ist eine CKD auch mit funktionellen und kognitiven Defiziten sowie Frailty assoziiert [2]. Patienten mit CKD sind zudem durch eine erhöhte Komplexität, gemessen u. a. an Komorbiditäten, gekennzeichnet [3].

Frailty beschreibt einen Zustand reduzierter Belastbarkeit und Widerstandsfähigkeit gegenüber Stressfaktoren. Ähnlich wie bei der Allgemeinbevölkerung ist Frailty bei CKD-Patienten mit ungünstigen klinischen Verläufen verbunden. Es können sowohl Assoziationen mit einer erheblich eingeschränkten Lebensqualität, einem erhöhten Sturzrisiko, vermehrten Hospitalisationen als auch mit einer erhöhten Mortalität gezeigt werden [4].

Eine einheitliche Definition von Frailty gibt es bisher nicht, es haben sich jedoch zwei Konzepte etabliert, denen sehr unterschiedliche Theorien zugrunde liegen. Der Phänotyp nach Fried charakterisiert Frailty als *Syndrom*, welches körperliche und funktionelle Einschränkungen beschreibt, die auf 5 verschiedenen Assessmentdomänen (körperliche Aktivität, Mobilität, Maß der Erschöpfung, Gewichtsverlust und Muskelkraft) basieren [5]. Das Modell der Defizit-Akkumulation definiert Frailty dagegen als das *Ergebnis einer Ansammlung funktioneller und klinischer Defizite*. Hier werden neben funktionellen Fähigkeiten auch Erkrankungen sowie die kognitive, psychische und soziale Situation miteinbezogen [6]. Zunehmend wird deutlich, dass Frailty als heterogenes Konstrukt komplexer ist als eine Kombination funktioneller Einschränkungen oder eine Akkumulation von Defiziten. Weiterentwicklungen rücken Frailty wieder näher an das Konzept eines beschleunigten Alterungsprozesses [7, 8].

Unabhängig von der Definition ermöglicht die Erkennung von Frailty bei Patienten mit CKD eine Identifizierung vulnerabler Patienten und damit die Entwicklung, Umsetzung und Überwachung gezielter Interventionen.

## Wie häufig ist Frailty bei Patienten mit CKD?

Die Prävalenz von Frailty bei Patienten mit CKD liegt deutlich höher als in der Allgemeinbevölkerung und ist weitgehend unabhängig vom Alter. Mehrere Studien zeigen eine Assoziation zwischen Abnahme der Nierenfunktion und Zunahme in der Prävalenz von Frailty [4].

Eine sehr hohe Prävalenz von Frailty findet sich bei dialysepflichtigen Patienten. Eine Analyse der Kohorte der US Renal Data System Dialysis Morbidity and Mortality Study identifizierte zwei Drittel von über 2000 Patienten als „frail“ (modifiziert nach Fried). Bemerkenswert ist, dass mehr als 60 % der 40- bis 60-Jährigen und 44 % der Patienten jünger als 40 Jahre als frail eingestuft wurden [9]. Auch in der Gruppe der Nierentransplantatempfänger liegt die Prävalenz von Frailty bei 20 % – bei einem durchschnittlichen Alter von 53 Jahren [10].

## Frailty-Screening bei CKD

Eine klare Empfehlung, welche Methode zur Beurteilung von Frailty bei Patienten mit CKD herangezogen werden sollte, gibt es aktuell nicht [11]. Unterschiedliche Screeningmethoden wurden bei CKD-Patienten evaluiert und sind jeweils unabhängig prädiktiv für eine erhöhte Morbidität und Mortalität, darunter der Phänotyp nach Fried, der Frailty-Index (FI), die Clinical Frailty Scale (CFS), der Groningen Frailty Indicator (GFI), die Edmonton Frailty Scale (EFS) und die FRAIL-Scale [12]. Die FRAIL-Scale bietet sich als einfacher „Bedside-Test“ in der ersten Einschätzung von Frailty an und zeigt bei Dialysepatienten eine gute Korrelation mit dialyseassoziierten Komplikationen [13].

**Figure 1:**
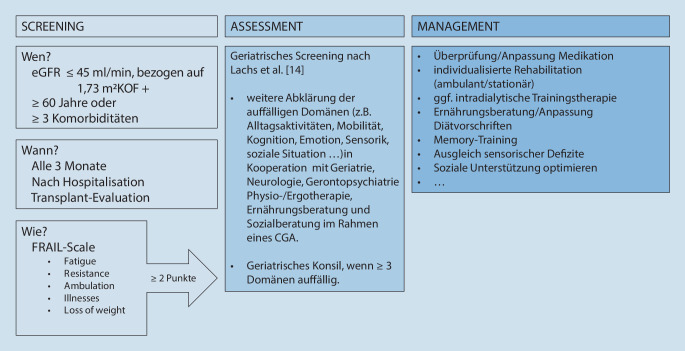

Der Identifikation eines Patienten als frail bzw. „prefrail“ sollte idealerweise eine weiterführende Abklärung mittels Comprehensive Geriatric Assessment folgen (Abb. 1). Um das Bewusstsein für Bereiche, die über Organspezifisches hinausgehen, in einem nichtgeriatrischen Setting zu schärfen, empfehlen wir das Screening nach Lachs et al. [14]. Für hier nicht oder nach neuen Erkenntnissen nicht ausreichend abgebildete Bereiche haben wir in Tab. 1 weitere Vorschläge gemacht.

**Table 1:** 

Begünstigende Faktoren	Assessmentvorschlag Fragen nach Lachs et al. [14] bzw. Alternative	Therapieoptionen
**Reduzierte Nahrungsaufnahme/Appetitlosigkeit** Urämietoxine Metabolische Acidose Chronische Inflammation Diätvorschriften	**„Rate a plate“ (**www.fightmalnutrition.eu**)** **Nutritional Risk Screen (NRS)**	**Lockerung der Diätvorschriften** **Ernährungsberatung** **Substitution von Bikarbonat (Ziel 22–24** **mmol/l)** *Cave: Polypharmazie*
**Verlust von Aminosäuren/Proteinen** Hämodialyse und Peritonealdialyse Urin (nephrotisches Syndrom)	**Eiweiß-Kreatinin-Quotient im Urin** **Serumalbumin**	**Ernährungsberatung** **Hochkalorische Nahrungsergänzung** **Kombination aminosäure- und glukosehaltiges Dialysat bei Peritonealdialyse **[40] **ACE-Hemmer/Sartane in Blutdrucktherapie bevorzugen** *Cave:* *Hypotonie*
**Erhöhte Aktivität kataboler Hormone** Parathormon Angiotensin II Kortikosteroide Glukagon	**–**	**–**
**Verminderte Aktivität anaboler Hormone** Insulin IGF‑1 Testosteron Vitamin D	**–**	**Substitution von Vitamin D 400–1000** **IE täglich (Zielspiegel 25-Hydroxy-Vitamin D >** **50–75** **nmol/l) **[41]
**Metabolische Acidose** Abbau von Muskelprotein Hemmung der Proteinsynthese Knochendemineralisierung Progress der CKD	**Blutgasanalyse** **Serumbikarbonat**	**Substitution von Bikarbonat (Ziel 22–24** **mmol/l)** *Cave: Polypharmazie*
**Chronic Kidney Disease – Mineral and Bone Disorder (CKD-MBD)** Osteopathie Erhöhte Frakturrate Extraossäre (vaskuläre) Kalzifizierungen	**Parathormon, Phosphat, Kalzium, 25-Hydroxy-Vitamin D, BAP (knochenspezifische alkalische Phosphatase)**	**Normale Phosphatwerte anstreben durch:** **– Anpassung Ernährung** *Cave: Mangelernährung* **– Phosphatbinder** *Cave: Polypharmazie* **Hyperkalzämie vermeiden** **Substitution von Vitamin D **[42]
**Volumenüberladung** Verminderte körperliche Belastbarkeit Chronische Inflammation	**–**	**Trinkmengenbeschränkung** *Cave:* *Hypotonie, Xerostomie/Dysphagie* **Kochsalzarme Ernährung** *Cave: Mangelernährung, Hypotonie* **Diuretische Therapie** *Cave: Elektrolytstörungen, Hypotonie, Polypharmazie*
**Anämie**	**Bestimmungen von Eisenstatus, Vitamin B**_**12**_** und Folsäure** **Ggf. Abklärung einer Blutung**	**Substitution von Eisen, Vitamin B**_**12**_** und Folsäure** **Transfusion und ESA nach individueller Nutzen-Risiko-Abwägung**
**Körperliche Inaktivität und Funktion** Erschöpfung Zeit an der Dialyse fehlt für Aktivität Kraftlosigkeit Sturzrisiko	**Aktivität: „Rapid Assessment of Physical Activity“ (RAPA) **[43] **Funktion: Gehgeschwindigkeit/ADL, Fragen nach Lachs et al**	**Individualisierte Rehabilitation (ambulant/stationär)** **Intradialytische Trainingstherapie**
**Komorbiditäten** Diabetes mellitus Kardiovaskuläre Erkrankungen Depression Demenz/Kognition	**Anamnese** **+** **Frage nach Lachs et al.** **+** **Basislabor** **Mini-Mental State Examination (MMSE)** **Uhrentest**	**Zielwerte für HbA**_**1c**_**, Blutdruck, LDL-Cholesterin individuell anpassen** **Gerontopsychiatrische Behandlung** **Memory Training, Ergotherapie**
**Polypharmazie** Arzneimittelinteraktionen, UAW	**Interaktionscheck** **+** **FORTA-Liste**	**Regelmäßige Überprüfung der Medikation, Anpassung an die Nierenfunktion**
**Sozialer Status**	**Fragen nach Lachs et al.**	**Sozialdienst einschalten** **Unterstützung organisieren**
**Sensorik** Hypakusis Sehschwäche	**Fragen nach Lachs et al.**	**Abklärung: HNO/Augenarzt** **Hörgerät, Brille/Lesehilfe anpassen**

### Zielgruppe und Screeningintervall von Frailty bei CKD

Die European-Renal-Best-Practice(ERBP)-Leitlinie zur Behandlung älterer Patienten mit CKD empfiehlt ein regelmäßiges Screening von funktionellem Status bzw. Frailty (alle 6 bis 8 Wochen bei Dialysepatienten bzw. bei jedem ambulanten Besuch) ab einem Alter von 65 Jahren und einer eGFR von < 45 ml/min, bezogen auf 1,73 m^2^ KOF [11]. Aufgrund der hohen Prävalenz von Frailty bei jüngeren CKD-Patienten scheint ein Screening jedoch auch unabhängig vom Alter gerechtfertigt. Ein zusätzliches Screening nach Hospitalisation und Operation oder im Rahmen der Evaluation für eine Nierentransplantation kann sinnvoll sein, um gezielte therapeutische Maßnahmen einzuleiten oder anzupassen [10]. Einen Vorschlag zum Screening haben wir Abb. 1 zusammengefasst.

## Ursachen von Frailty bei CKD

**Figure 2:**
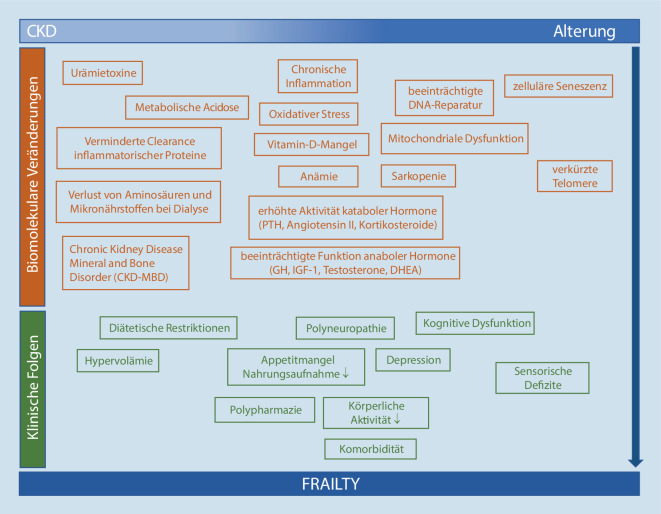

Die Ursachen von Frailty sind multifaktoriell. Neben altersbedingten biomolekularen Veränderungen (Inflammaging, veränderter Hormonstatus, Immunseneszenz) können psychologische (Stress, Depression), genetische und soziale Faktoren sowie chronische Erkrankungen zur Entwicklung von Frailty beitragen und sich gegenseitig verstärken [15]. Die chronische Nierenerkrankung, häufig verbunden mit anderen chronischen Erkrankungen und Polypharmazie, hat deshalb einen wesentlichen Einfluss auf die Entwicklung von Frailty. Es wundert nicht, dass sich Biomarker für Frailty und beschleunigte Alterungsprozesse mit Prognosemarkern von CKD überlappen [16]. Die begünstigenden Faktoren für Frailty sind in Abb. 2 zusammengefasst.

### Mangelernährung und „protein-energy wasting“

Die International Society of Renal Nutrition and Metabolism (ISRNM) prägte den Begriff Protein-energy wasting (PEW) als Zustand verringerter Protein- und Energiereserven bei Patienten mit CKD [17]. Die Ursachen von PEW und Frailty überlappen und führen zu einer engen Assoziation der beiden Syndrome.

Bereits in frühen CKD-Stadien lässt sich eine Verminderung der Protein- und Energieaufnahme nachweisen; Appetitlosigkeit besteht schließlich bei etwa einem Drittel aller Dialysepatienten [18, 19]. Daneben bestehen bei Patienten mit CKD eine Vielzahl von Diätvorschriften, welche die Aufrechterhaltung einer ausreichenden Proteinzufuhr erschweren.

### Chronische Inflammation, oxidativer Stress und metabolische Acidose

Mit Abnahme der Nierenfunktion kommt es über mehrere Mechanismen zu einem Anstieg proinflammatorischer Zytokine, u. a. Interleukin‑6 (IL-6) und Tumor-Nekrose-Faktor α (TNF-α) [17]. Diese chronische systemische Inflammation wird durch oxidativen Stress (Zunahme freier Sauerstoffradikale und „advanced glycation end products“) sowie eine metabolische Acidose verstärkt. Dieses entzündliche Milieu dämpft den Appetit, steigert den Grundumsatz und fördert den Abbau von Muskelprotein über das Myostatin‑, Caspase-3- und Ubiquitin-Proteasom-System [20]. Ein daraus resultierendes Ungleichgewicht von Energieaufnahme und Energieverbrauch ist wesentlich in der Entwicklung von PEW, Sarkopenie und Frailty.

Zudem sind zelluläre Seneszenz, Verlust von Telomerstrukturen, mitochondriale Dysfunktion und schlechte DNA-Reparaturfähigkeit am Alterungsprozess und der Entwicklung von Frailty beteiligt. Diese Prozesse treten bei CKD vorzeitig und beschleunigt auf und sind vermutlich ein Ergebnis des urämischen Milieus [17].

Insgesamt entspricht eine CKD auf molekularer Ebene einem Zustand beschleunigter metabolischer Alterungsprozesse, was das Auftreten von Frailty auch bei jüngeren CKD-Patienten erklären kann.

### Körperliche Aktivität

Niedrige Spiegel von 25-Hydroxy-Vitamin‑D sind bei älteren Menschen mit einem erhöhten Risiko für Sarkopenie und Frailty assoziiert. Mit zunehmender Niereninsuffizienz kommt es zu einem Mangel an 1,25-Dihydroxy-Vitamin‑D, welcher mit einer reduzierten körperlichen Leistungsfähigkeit und Abnahme der Muskelmasse bei Patienten mit CKD assoziiert ist [21].

Körperliche Aktivität ist bei Patienten mit CKD eingeschränkt und nimmt mit Verschlechterung der Nierenfunktion weiter ab. Dialysepatienten sind, verglichen mit einer gesunden, aber bewegungsarmen Bevölkerungsgruppe, 35 % weniger aktiv [22].

## Management von CKD-Patienten mit Frailty

Da Frailty in der Ausprägung multidimensional ist, muss auch ein therapeutischer Ansatz mehrere verschiedene Bereiche adressieren und interdisziplinär angelegt sein. Grundlage für Interventionen sollte immer eine ganzheitliche Beurteilung des Gesundheitszustandes, der Funktionalität und des sozialen Umfelds durch ein Comprehensive Geriatric Assessment sein [23, 24]. Allgemeine Empfehlungen zu Prävention und Management von Frailty sind in der European Perspective of Joint Action on Frailty und den ICFSR International Clinical Practice Guidelines ausführlich beschrieben [25, 26]. Einen Überblick über CKD-spezifische Optionen unter Berücksichtigung der oben genannten allgemeinen Empfehlungen bietet Tab. 1.

### Therapie renaler Folgeerkrankungen und relevanter Komorbiditäten

Die eingeschränkte Nierenfunktion ist wesentlich an der Entwicklung von Frailty beteiligt, und eine frühzeitige Behandlung renaler Folgeerkrankungen kann ein wichtiger Baustein im Management und in der Prävention von Frailty sein. Therapiestrategien für die generelle CKD-Population sind jedoch nicht ohne Weiteres auf gebrechliche Patienten übertragbar. Trotz der wachsenden Zahl älterer und gebrechlicher Patienten mit CKD mangelt es an hochwertigen, prospektiven Studien in dieser Patientengruppe. Aufgrund der Heterogenität, sowohl in der Ausprägung als auch in den zugrunde liegenden Faktoren von Frailty, sind zudem eine Individualisierung der Therapie und Einbindung der Patienten im Hinblick auf Therapieziele notwendig.

Die ERBP-Leitlinie hat dies adressiert und gibt u. a. Empfehlungen zur Bestimmung der Nierenfunktion im Alter, zum Screening und zu therapeutischen Optionen von Mangelernährung und funktionellen Defiziten sowie Entscheidungshilfen bezüglich der Einleitung einer Dialyse [11]. Die Behandlungen renaler Folgeerkrankungen und relevanter Komorbiditäten werden jedoch nicht angesprochen.

Wir schließen uns hier der Empfehlung anderer Autoren an, den Frailty-Status in die Therapieentscheidungen miteinzubeziehen und individuell zu beurteilen, ob Abweichungen von empfohlenen Zielwerten (z. B. für Bikarbonat, Phosphat, HbA_1c_, LDL-Cholesterin, Blutdruck, Hämoglobin) angestrebt bzw. toleriert werden sollten [27].

Die Behandlung einer metabolischen Acidose (Serumbikarbonat ≤ 22 mmol/l) verbessert z. B. Ernährungszustand und Muskelkraft und kann den CKD-Progress verlangsamen, erhöht jedoch die Tablettenlast [28].

Die neue KDIGO-Guideline zum Management von Diabetes bei CKD empfiehlt eine Individualisierung von HbA_1c_-Zielen mit Akzeptanz höherer Zielwerte bis 8 %, u. a. je nach Schweregrad der CKD, Anzahl an Komorbiditäten und Lebenserwartung [29].

### Beurteilung der Nierenfunktion und Überprüfung der Medikation

Polypharmazie (Einnahme ≥ 5 Medikamente) trägt unabhängig von Komorbiditäten zur Entwicklung von Frailty bei [30]. Dialysepatienten nehmen durchschnittlich 10 bis 12 verschiedene Medikamente und im Mittel 19 Tabletten/Tag ein [31]. Polypharmazie, die Verschreibung inadäquater Medikamente oder falsche Anpassung der Dosis an die Nierenfunktion sind bei älteren Patienten mit CKD häufig und fordern eine regelmäßige Überprüfung der Medikation. Hilfestellung bieten hier die STOPP – START- oder die FORTA-Kriterien [32, 33].

Zur Anpassung der Medikamentendosis an die Nierenfunktion älterer und/oder gebrechlicher Patienten ist die alleinige Beurteilung des Kreatininwerts nicht aussagekräftig. Verwendet werden sollten die GFR-Annäherungsformeln, wobei die CKD-EPI_Krea-Cys_- gegenüber der CKD-EPI_Krea_-Formel Vorteile hat [11]. Die Cockcroft-Gault-Formel zeigt ebenfalls gute Korrelation mit der Nierenfunktion bei älteren Patienten, insbesondere bei Sarkopenie [11]. Die speziell zur Abschätzung der Nierenfunktion bei älteren Menschen entwickelten BIS-1- und BIS-2-Formeln zeigen bei Patienten ≥ 70 Jahren eine gute Korrelation mit der direkt gemessenen GFR [34].

### Ernährung

Das Risiko einer Mangelernährung kann bei strengen Diätvorschriften für ältere und gebrechliche Dialysepatienten höher wiegen als der potenzielle Nutzen. Die ERBP-Leitlinie empfiehlt daher, dass „die Erhaltung eines adäquaten Ernährungszustandes gegenüber allen anderen Diätvorschriften Vorrang hat“ [11].

Ein Cochrane-Review konnte zeigen, dass eine hochkalorische Nahrungsergänzung bei älteren Patienten mit CKD zu einer geringen, aber beständigen Gewichtszunahme beiträgt [35]. Der Einsatz einer parenteralen intradialytischen Ernährung wird kontrovers diskutiert, nicht zuletzt, weil ein Vorteil gegenüber einer oralen Nahrungsergänzung bisher nicht nachgewiesen werden konnte [11].

### Bewegung/Training

Sowohl eine Verbesserung von Muskelkraft, Gehstrecke, funktionellen Fähigkeiten und gesundheitsbezogener Lebensqualität konnte unter körperlichem Training gezeigt werden [36]. Bei dialysepflichtigen Patienten ist regelmäßiges Training an Nichtdialysetagen am effektivsten, intradialytisches Training wird von Patienten jedoch besser angenommen [37]. Seit April 2019 rekrutiert zudem die Studie Dialyse-Trainings-Therapie (DiaTT) in Deutschland. Diese Studie untersucht an geplanten 1100 Patienten die Effektivität einer Trainingstherapie während der Dialyse, welche auch den Frailty-Status berücksichtigt (clinicaltrials.gov NCT03885102). Aus der klinischen Erfahrung kann gerade bei gebrechlichen Patienten eine Dialyse am Nachmittag sinnvoll sein, um am Vormittag eine Trainingseinheit zu ermöglichen, welche nach der Dialyse oft nicht mehr leistbar ist.

### Dialyse

Da das urämische Milieu an der Entstehung von Frailty bei Patienten mit CKD beteiligt ist, wäre unter pathophysiologischen Gesichtspunkten zu erwarten, dass die Dialyse zu einer Verbesserung des Frailty-Status führt. Die bisherige, spärliche Datenlage zeichnet hier jedoch ein anderes Bild.

Eine prospektive Studie untersuchte die Entwicklung von Frailty und funktionellem Status nach Dialysebeginn bei 187 Patienten ≥ 65 Jahren, die bisher selbstständig zu Hause lebten. Sechs Monate nach Dialysebeginn konnten 34 % den funktionellen Status halten, bei 18 % kam es zu einer Verbesserung, und bei 40 % kam es zu einer Verschlechterung des funktionellen Status. Das Risiko für eine Verschlechterung war besonders hoch für als frail eingestufte Patienten, unabhängig von Alter sowie Patienten ≥ 75 Jahre [38].

Prospektive Studien, welche eine Dialyse mit einer konservativen Therapie bei älteren und/oder gebrechlichen Patienten vergleichen, existieren bisher nicht. Die vorhandenen Daten stammen aus Beobachtungsstudien, in denen Frailty selten evaluiert wurde. Laut ERBP-Leitlinie beobachtet man bei „ausgewählten“ älteren und gebrechlichen Patienten nach Beginn einer Dialyse einen Überlebensvorteil von einem bis 2 Jahren gegenüber einer konservativen Therapie, auch wenn sich dieser bei Patienten mit vielen Komorbiditäten deutlich abschwächte. Die Lebensqualität unterschied sich in beiden Patientengruppen nicht wesentlich [11].

Eine Aufklärung von Nephrologen und Geriatern über Nierenersatztherapien sollte daher bei älteren und gebrechlichen Patienten eine genaue Evaluation von Lebens- bzw. Therapiezielen beinhalten und eine konservative Therapie als Alternative zur Dialyse erläutern. Je gebrechlicher Patienten sind und je mehr Komorbiditäten vorliegen, umso weniger scheinen diese von einer Dialyse zu profitieren. Zur Abschätzung der Mortalität bei Patienten mit terminaler Niereninsuffizienz kann der REIN-Score als kumulativer Score über das Vorhandensein relevanter Komorbiditäten hilfreich sein und in die Beratung einbezogen werden [11].

### Nierentransplantation

Zwar konnte für präoperativ als frail eingestufte Patienten ein erhöhtes Risiko für eine verzögerte Funktionsaufnahme der Transplantatniere, eine Wiederaufnahme in ein Krankenhaus, Komplikationen im Rahmen der Immunsuppression und Mortalität gezeigt werden [10]. Nachdem es postoperativ zu einer Verschlechterung des Frailty-Status kommt, verbessern jedoch die meisten Patienten langfristig ihren Frailty-Status [10].

### Pharmakotherapie

In einer kleineren Studie bei Patienten mit CKD und Vitamin-D-Mangel konnte eine Vitamin-D-Substitution zu einer verbesserten körperlichen Belastbarkeit führen [20]. Daneben wird der Einsatz anaboler Hormone wie Testosteron, somatotropes Hormon (GH) und IGF‑1 bei Patienten mit Frailty und CKD untersucht, wobei endgültige Daten zu Wirksamkeit und Sicherheit bisher fehlen [17].

## Ausblick und offene Fragen

Studien zu CKD und Frailty aus Deutschland gibt es bisher kaum, und dies obwohl in Deutschland 65 % der Dialysepatienten ≥ 65 Jahre und etwa 18 % der Dialysepatienten ≥ 80 Jahre alt sind [39]. Diese Studien sind notwendig, um eine optimale Versorgung älterer und gebrechlicher CKD-Patienten zu erbringen. Die Erkennung von Frailty und den beteiligten Faktoren durch ein geriatrisches Assessment kann dazu beitragen, patientenzentrierte Therapiestrategien in dieser komplexen Patientengruppe zu entwickeln.

Offen Fragen derzeit sind:

- Welche Methode ist am besten geeignet, Frailty in CKD-Patienten zu erkennen und Komplikationen vorherzusagen?
- Kann eine frühzeitige nephrologische Mitbetreuung bei Patienten mit CKD das Entstehen bzw. Fortschreiten von Frailty beeinflussen?
- Welche therapeutischen Interventionen sind für die Behandlung von Frailty bei CKD-Patienten wirksam und für die Patienten bedeutsam?
- Kann der Frailty-Status hilfreich bei der Entscheidung Dialyse vs. konservative Therapie sein?
- Gibt es eine überlegene Dialyseform für gebrechliche Patienten?

Zusammenfassend empfehlen wir auf Basis der Empfehlungen der ERBP-Leitlinie und der Empfehlungen der geriatrischen Gesellschaften eine für den deutschen Raum konsentierte Empfehlung zu entwickeln, wie Frailty bei CKD-Patienten im niedergelassenen und klinischen Setting erkannt, weiterdiagnostiziert und behandelt werden sollte.

## Fazit für die Praxis

- Frailty tritt bei Patienten mit CKD früher und häufiger auf als in der Allgemeinbevölkerung.
- Frailty ist ein wichtiger und potenziell modifizierbarer Risikofaktor für eine erhöhte Sterblichkeit und Hospitalisationsrate sowie eine reduzierte Lebensqualität.
- Zur Messung von Frailty existieren viele verschiedene Instrumente, wobei bisher keines in der Erkennung von Frailty bei CKD als überlegen identifiziert worden ist.
- Die Auswahl des Screeninginstruments soll unter Berücksichtigung der klinischen Rahmenbedingungen und der verfügbaren Ressourcen, einschließlich Zeit und Personal, erfolgen.
- Die Genese von Frailty ist multifaktoriell, und direkte Folgen der CKD tragen wesentlich zur Entstehung bei.
